# Listening to the Mind: Integrating Vocal Biomarkers into Digital Health

**DOI:** 10.3390/brainsci15070762

**Published:** 2025-07-18

**Authors:** Irene Rodrigo, Jon Andoni Duñabeitia

**Affiliations:** Centro de Investigación Nebrija en Cognición (CINC), Universidad Nebrija, 28015 Madrid, Spain; irodrigh@nebrija.es

**Keywords:** vocal biomarkers, singing, digital health, mental health, neurodegenerative diseases

## Abstract

The human voice is an invaluable tool for communication, carrying information about a speaker’s emotional state and cognitive health. Recent research highlights the potential of acoustic biomarkers to detect early signs of mental health and neurodegenerative conditions. Despite their promise, vocal biomarkers remain underutilized in clinical settings, with limited standardized protocols for assessment. This Perspective article argues for the integration of acoustic biomarkers into digital health solutions to improve the detection and monitoring of cognitive impairment and emotional disturbances. Advances in speech analysis and machine learning have demonstrated the feasibility of using voice features such as pitch, jitter, shimmer, and speech rate to assess these conditions. Moreover, we propose that singing, particularly simple melodic structures, could be an effective and accessible means of gathering vocal biomarkers, offering additional insights into cognitive and emotional states. Given its potential to engage multiple neural networks, singing could function as an assessment tool and an intervention strategy for individuals with cognitive decline. We highlight the necessity of further research to establish robust, reproducible methodologies for analyzing vocal biomarkers and standardizing voice-based diagnostic approaches. By integrating vocal analysis into routine health assessments, clinicians and researchers could significantly advance early detection and personalized interventions for cognitive and emotional disorders.

## 1. Introduction: Vocal Biomarkers

This Perspective argues that vocal biomarkers can provide valuable information for the diagnosis and symptom tracking of cognitive and mental health conditions. We propose that singing could substitute speech as the standard source of voice features, as it may enhance engagement and compliance, especially in populations with linguistic or cognitive impairments, while still offering rich acoustic parameters for analysis. Developing standardized, singing-based vocal assessments may improve the sensitivity of early screening for dementia and mental health conditions, paving the way for novel diagnostic and therapeutic applications.

The human voice conveys critical information and plays a central role in social interaction. Beyond enabling communication, the voice allows individuals to express emotions, intentions, stress, and contextual nuances by modulating tone and pitch [1]. Considering that the voice requires the coordination of various cognitive and motor processes, even small changes in it can provide a sensitive snapshot of cognitive functioning, relevant to many diseases and mental states [2]. These changes, or vocal biomarkers, refer to quantifiable voice characteristics that reflect underlying physiological and psychological states. They are a non-invasive tool that can detect subtle neurocognitive and emotional variations that may go unnoticed in traditional assessments [3]. Compared to conventional diagnostic methods, vocal biomarkers excel in their ability to detect early indicators of conditions such as depression, anxiety, and neurodegenerative diseases [4,5,6].

Within this framework, vocal biomarkers have the potential to effectively distinguish between healthy individuals and those experiencing cognitive or emotional impairments [7]. Still, systematic and rigorous evaluation of digitally obtained voice biomarkers is crucial to ensure that they provide accurate measurement and can serve as a suitable alternative for detecting and monitoring neurodegenerative diseases and mental health conditions [8]. Recent research leveraging machine learning and automated speech analysis techniques has demonstrated that acoustic features are effective in predicting the severity of depression and suicidal tendencies based on short audio recordings [9]. Additionally, the ability to passively collect vocal biomarkers through smartphones and wearables makes them an accessible and scalable solution for remote health monitoring, especially in populations with limited access to specialized care. Given the rapid advancements in artificial intelligence (AI) and digital health, it is imperative to integrate vocal biomarkers into routine clinical assessments to enhance diagnostic accuracy and enable early intervention.

## 2. Vocal Biomarkers: Acoustic and Linguistic Features

Vocal biomarkers are derived from both acoustic and linguistic features of spoken language. Among the **acoustic features**, prosodic features such as pitch, formants, energy, jitter, and shimmer are foundational. Pitch can indicate alterations in emotional state or neurodegenerative changes [10], formants are critical for vowel articulation [11], and jitter and shimmer are often elevated in conditions affecting motor coordination or vocal fold control [10]. Other acoustic features of relevance are spectral features such as the spectral flux, slope, centroid, entropy, roll-off, and flatness, which offer a complementary dimension, capturing how energy is distributed across frequencies [10]. Measures of voice quality further enrich the acoustic features and profile. For example, the zero-crossing rate indicates the clarity of the voice [12], and the harmonic-to-noise ratio (HNR) and its inverse, the noise-to-harmonic ratio (NHR), reflect the periodicity and breathiness of the voice, often disrupted in neurodegenerative diseases [10].

On the linguistic side, biomarkers are extracted from transcribed speech or natural language processing (NLP) analyses of spoken output. These include **linguistic features** associated with lexical richness, such as vocabulary diversity, word length, and word frequency usage, which are known to correlate with cognitive reserve and verbal fluency [13]. Additionally, syntactic complexity can reflect working memory load and executive functioning [14]. While the aim of the current article is not to provide a fully detailed picture of the association between all the vocal biomarkers with all possible conditions, in Table 1, we summarize the most relevant vocal biomarkers, indicating some of the conditions they are associated with, with a special focus on stress, major depressive disorder (MDD), neurodegenerative conditions such as Alzheimer’s disease (AD), Parkinson’s disease (PD), frontotemporal dementia (FTD), and other psychiatric conditions such as bipolar disorder type I (BDI).

Some voice biomarkers have been more studied than others, as shown in Table 1. F0, jitter, shimmer, speech rate, and speech pause duration are among the most studied ones, and they have already been established as reliable vocal biomarkers for a variety of diseases, including stress [11,15,16,17,18], depression [11,19,20], PD [21,22], and dementia [13,23]. On the other hand, some other voice biomarkers, and particularly those relying on spectral features, have not yet accumulated a substantial amount of supportive data. Still, these spectral measures appear to be more reliable than traditional features across varying levels of impairment severity [10] and have shown promise in distinguishing between normal aging and pathological conditions [24]. Thus, even though vocal biomarkers have proven valuable in the assessment of several pathologies, they are not yet formally used in clinical or real-world practice. The field of vocal biomarkers is still emerging, and widespread clinical adoption remains a distant goal. Nonetheless, the existing evidence supports an integrative assessment approach that includes vocal biomarkers as an additional non-invasive source of diagnostic information.

The choice of features is dictated by the specific disorder or cognitive condition under investigation and by the nature of the voice sample, whether it is prompted or spontaneous, scripted or conversational, short or extended, as both acoustic and linguistic features present advantages or disadvantages depending on the circumstances. Linguistic features that rely heavily on semantic meanings do not perform well in analyzing structured conversations, while acoustic features tend to be more reliable in structured conversations, as well as in capturing emotional changes [25]. Studies analyzing linguistic features generally achieve slightly higher performance levels due to their richness in information. However, these features need a more in-depth analysis of discourse content, and they usually require automatic speech recognition (ASR) before analysis of the features can be conducted, which affects the robustness of the study. In addition, they must also consider the uniqueness of the language; therefore, these algorithms are designed for specific languages [26]. As a result, linguistic features may have limitations in transferability. In contrast, acoustic features are independent of linguistic content and have a strong transferability. Therefore, algorithms focusing on acoustic features demonstrate universality, allowing researchers from diverse countries to study various languages and speech-eliciting tasks [26].

Hence, combining acoustic and linguistic features seems to provide both higher accuracy and higher universality. In support of this idea, Tang et al. [15] analyzed both acoustic and linguistic features of healthy adults and MCI in a semi-structured conversational setting, which can be used outside clinical settings. They obtained higher predictability in the model that combined both types of features. Still, the available integrative data are scarce, and more research on this topic is needed to draw conclusions. The ongoing integration of voice analytics with machine learning can enable precise and individualized profiling of cognitive and mental health across the lifespan.

**Table 1:** Most relevant voice biomarkers, their classification, basic definition, and conditions they are associated with.

Classification	Sub-Classification	Feature	Definition	Condition
Acoustic features	Prosodic	Pitch	Reflects the fundamental frequency (F0) of vocal fold vibration [16].	Stress [11,15,16,18], MDD [19,20], BDI [11], PD [21], AD [27], cognitive impairment [23].
		Formants (F1, F2)	The first and second peaks in the spectrum that result from a resonance of the vocal tract [11].	Stress [11,16], cognitive impairment [23].
		Jitter	Measure of variation in frequency [10].	Stress [18], MDD [11], AD [27], PD [21,22].
		Shimmer	Measure of variation in amplitude [10].	Stress [15,17], MDD [11], BDI [11], PD [21,22].
		Energy	Represents the intensity [11].	Stress [18], MDD [11].
	Spectral features	Spectral Centroid	Brightness and sharpness of sound [24].	Stress [17], PD [24].
		Spectral Spread	Standard deviation around the spectral centroid [24].	PD [24].
		Spectral Flux	The rate of change of the spectrum [24].	PD [24].
		Spectral Flatness	Capture the presence of a large number of peaks in the spectrum [24].	PD [24].
		Spectral Kurtosis	Measure of the flatness of the spectrum distribution around its mean value [24].	PD [24].
		Spectral Skewness	Measure of the spectrum distribution asymmetry around its mean value [24].	PD [24].
	Voice quality	Zero-crossing rate	Rate at which the signal changes from positive to negative, or vice versa [12].	MDD [12].
		Harmonic-to-noise ratio (HNR)	Turbulent noise present in the voice signal [10].	Stress [16], MDD [11], PD [22].
		Noise-to-harmonic ratio (NHR)	The opposite of HNR [10].	Stress [16].
	Articulatory features	Mel-frequency cepstral coefficients (MFCCs)	Power density spectrum of speech, presented on a frequency scale [22].	Stress [17], MDD [11], BDI [11], PD [22].
Linguistic features	Lexical	Vocabulary diversity	Complexity of vocabulary [13].	Dementia (AD, FTD) [13] and PD [28].
		Word length	Average length of words used [13].	Dementia (AD, FTD) and cognitive impairment [13].
		Word frequency usage	For instance, noun or adjective frequency [13].	Dementia (AD, FTD) [13] and PD [28].
	Syntactic	Syntactic complexity	The usage of different syntactic structures and measures of syntactic complexity [13].	Dementia (AD, FTD) [13] and PD [28].
	Temporal	Speech rate	Words per minute [13].	Stress [18], MDD [11], BDI [11], dementia (AD, FTD) [13], PD [22,28], and cognitive impairment [23].
		Speech pause duration	Duration of pauses taken during speaking, both between and within words [22].	Stress [15,16], MDD [11], dementia (AD, FTD) [13], PD [22,28], and cognitive impairment [23].

## 3. Vocal Biomarkers in Mental and Emotional Health

Vocal biomarkers have shown promise in diagnosing emotional states, as vocal expression is deeply intertwined with psychological well-being. Techniques for extracting prosodic, spectral, and articulatory features from voice recordings have direct applications in detecting conditions such as major depressive disorder, stress, and generalized anxiety disorder [11,29,30]. For instance, research has demonstrated that heightened stress levels correlate with increased F0, altered formant structures, and increased breathiness due to glottal waveform changes [16].

To date, there is no available standard biomarker for the detection of mental health conditions, and often, the tracking of symptoms relies primarily on patient self-report. This method, while accessible and low-cost, is subject to recall bias, underreporting, and variability in self-awareness or willingness to disclose symptoms, especially in populations such as adolescents, older adults, or individuals with cognitive impairments. Notably, stress-related symptoms are among the most frequently underreported, despite their profound impact on physical and mental health [31]. The lack of objective, physiological indicators for emotional states presents a major barrier to early diagnosis and effective treatment. In this context, passive voice monitoring offers a compelling alternative. By analyzing speech continuously or at high frequency, without requiring active input from the user, it becomes possible to detect subtle vocal changes associated with emotional strain and mental health conditions [32]. Vocal features such as pitch, jitter, shimmer, energy, MFCC, and HNR can be used to detect changes in emotional content [33].

High-frequency patient monitoring using voice data has shown promise in the early identification and prediction of several mental health conditions, including self-harm, suicide risk, depression, bipolar disorder, schizophrenia, and substance abuse [29]. These disorders often present with prodromal or episodic changes in voice that are difficult to capture through traditional clinical encounters. Artificial Intelligence-driven voice analysis leverages machine learning algorithms to detect these subtle, often imperceptible, vocal patterns. Recent studies have demonstrated that features such as monotonic prosody, increased vocal effort, and reduced verbal fluency are reliable indicators of affective disturbances. For instance, a recent study by Zhang et al. [34] showed that short speech samples analyzed through automated systems could significantly improve depression screening accuracy, even when compared with established questionnaire-based methods. Likewise, AI models trained on large voice datasets can differentiate between anxiety and depressive symptoms based on acoustic and linguistic cues [1]. These findings highlight the potential of vocal biomarkers as scalable tools for mental health monitoring, with applications in clinical, educational, and telehealth settings. Ultimately, the integration of voice analysis into digital health applications could support real-time, personalized interventions, reducing the burden on healthcare systems and offering continuous care for individuals in need.

A common finding across multiple studies is the capacity of vocal features to reflect internal psychological states without reliance on linguistic content. For example, Namkung et al. [17] demonstrated that stress-induced changes in healthy individuals could be detected through acoustic features alone. Using the ECAPA-TDNN deep learning model, the study analyzed voice recordings collected before and after stress induction via the Socially Evaluated Cold Pressor Test (SECPT). The model achieved 70% accuracy, a substantial achievement considering the complexity and variability of emotional expression across individuals. These results are particularly compelling because they demonstrate that stress, conceived as an internal, subjective state, is detectably encoded in the acoustic signature of the voice. As such, this study underscores the viability of voice as a real-time, non-invasive diagnostic aid, particularly in settings where self-reporting is impractical or biased.

Complementing this, Di et al. [19] focused on persistent affective disorders, namely MDD, in a large-scale study involving thousands of participants across multiple clinical sites. They found consistent associations between depression and reduced vocal pitch as well as flattened pitch modulation. These acoustic markers were identified in a large multisite case-control study, enhancing the external validity of the findings. Notably, even individuals with a genetic predisposition to MDD, but no active symptoms, exhibited similar vocal traits, suggesting a dual utility of certain vocal biomarkers: as indicators of current mental health status and as potential markers of latent vulnerability or predisposition. Hence, the utility of vocal biomarkers goes beyond mere symptom tracking, also providing value for early identification.

Despite promising results, these studies used data from controlled environments; the study by Namkung et al. [17] used real-time data, but with artificial stress conditions, while the study by Di et al. [19] used data from a previous study, altogether raising the question of whether vocal biomarkers are applicable in real-world settings and how accurate they are. In this line, Larsen et al. [18] contributed to the growing evidence supporting the longitudinal utility of vocal biomarkers. Their prospective cohort study in outpatient psychiatric settings aimed to validate a composite vocal score incorporating features such as jitter, shimmer, pitch, energy variability, vowel space, speech rate, and pause duration. The score dynamically tracked fluctuations in mental health status over time and remained robust under real-world conditions, showing that acoustic biomarkers retain diagnostic relevance even amid the background noise and variability of everyday life.

Together, these studies reflect a broader trend: vocal biomarkers are emerging as a scalable and cost-effective complement to conventional diagnostic tools. While their individual applications vary, they collectively highlight the substantial potential of integrating vocal biomarkers into digital health solutions for the early detection, continuous monitoring, and timely intervention of mental health conditions. By enabling passive, unobtrusive, and frequent assessments, voice-based technologies can support personalized care strategies and reduce reliance on episodic clinical visits.

## 4. Vocal Biomarkers and Cognitive Health

Importantly, the applications of vocal biomarkers extend beyond emotional and psychiatric assessment. Increasing evidence suggests that voice can also serve as a sensitive marker of neurodegenerative diseases, offering a non-invasive and scalable means to detect early signs of cognitive decline. Changes in speech fluency, articulation, prosody, and lexical access have been observed in the prodromal phases of conditions such as Alzheimer’s disease and other forms of dementia [35]. These alterations, often too subtle for caregivers or even clinicians to detect early on, can be systematically captured and analyzed using automated voice processing tools. Early detection is especially critical in the case of dementia, as current pharmacological and behavioral treatments are most effective when implemented during the initial stages of the disease, before significant neurodegeneration has occurred [36]. As such, vocal biomarkers could play a transformative role in enabling proactive intervention and delaying functional decline, especially in aging populations where early diagnosis remains a persistent challenge.

Monitoring cognitive health is crucial for the early diagnosis and timely intervention of dementia, as therapeutic efficacy is highest during the initial stages of neurodegenerative progression. A wide range of screening methods, including brain imaging techniques (such as MRI and PET scans), blood-based biomarkers, and cerebrospinal fluid (CSF) analyses, have been developed to detect early pathological changes associated with conditions like AD. However, despite their diagnostic accuracy, these approaches are often invasive, costly, and logistically demanding, limiting their feasibility for routine or large-scale population screening, especially in asymptomatic individuals or those with only mild cognitive concerns [37]. In this context, vocal biomarkers have emerged as a promising, non-invasive, and scalable alternative for detecting early cognitive impairments. Speech is a complex behavior that relies on the coordinated functioning of multiple neural systems involved in memory, attention, executive control, and motor planning. Subtle alterations in speech timing, fluency, prosody, and lexical retrieval have been consistently reported as some of the earliest signs of neurodegenerative diseases such as AD and PD [13,28]. While these early-stage vocal irregularities may go unnoticed by casual observers or even clinicians, they can be systematically captured and analyzed through automated voice analysis tools using acoustic and linguistic feature extraction [28,38].

In this light, vocal biomarkers serve not only as a cost-effective complement to traditional screening methods but also as an innovative approach to studying the subclinical stages of cognitive decline, where symptoms are subtle and often missed in standard clinical assessments. This is especially relevant in Parkinson’s disease, where symptoms become clinically evident only after 60–80% of dopamine-producing neurons have already been destroyed, making early detection essential to slowing disease progression [21]. Speech and voice alterations are among the most prevalent and earliest observable symptoms of PD. In fact, nearly 90% of individuals with PD experience some form of speech disorder [39], and as many as almost 80% of patients with early-stage PD already present detectable changes in voice features [1]. These impairments are typically categorized under hypokinetic dysarthria, a motor speech disorder characterized by monotone prosody, reduced pitch variability (F0), shimmer, imprecise articulation, and diminished overall speech clarity [1,24]. Importantly, a reduction in F0 variability during spontaneous speech has been observed up to five years before clinical diagnosis, reinforcing the potential of vocal analysis as a preclinical diagnostic tool [40]. Recently developed machine learning models trained to analyze phonation and articulation patterns have shown high sensitivity in detecting early PD symptoms. For instance, Tracy et al. [23] implemented a machine learning-based framework that successfully distinguished early PD cases based on acoustic features, offering a promising, scalable tool for early diagnosis and monitoring in routine clinical practice or remote care settings. Similarly, in AD, early cognitive declines often go unnoticed until more pronounced deficits emerge, usually several years after the onset of underlying pathology [41]. However, subtle linguistic markers, such as average syllable duration, articulation rate, and speech rate, can precede formal diagnosis and are commonly observed in individuals at the Mild Cognitive Impairment (MCI) stage [42].

Beyond lexical markers, prosodic features such as pitch, pulse, and jitter have demonstrated diagnostic value in distinguishing between normal aging and MCI [2,42,43]. Moreover, longitudinal research has shown that participants who were later diagnosed with dementia already exhibited increased hesitations and pause frequency during spontaneous speech at a stage when no formal symptoms were yet recognized [27]. These findings suggest that vocal and linguistic analysis could offer a valuable, non-invasive means of detecting neurodegeneration years before clinical diagnosis, opening new pathways for early intervention and monitoring of disease progression.

Recent studies have increasingly focused on evaluating whether acoustic features of speech can serve as reliable biomarkers to differentiate individuals with cognitive impairment from healthy controls. In a large-scale investigation, Nagumo et al. [44] analyzed speech samples from 8779 participants with varying cognitive profiles, classified according to their scores on the Mini-Mental State Examination (MMSE). Participants were grouped into healthy controls, individuals with Mild Cognitive Impairment, and those with Global Cognitive Impairment (GCI), defined by MMSE scores ranging from 20 to 23. To assess the discriminative power of speech, a machine learning model was employed to analyze a range of acoustic features, including formant frequencies (F1 and F2), fundamental frequency, number and duration of pauses, syllable duration, and total utterance duration. These features reflect both the motor and cognitive components of speech production, which are often compromised in individuals with cognitive decline. The results demonstrated that the model could successfully differentiate individuals with MCI and GCI from healthy controls with notable accuracy, underscoring the diagnostic potential of vocal biomarkers in identifying early cognitive changes.

## 5. The Role of Singing in Cognitive and Emotional Assessment and Training

Most studies exploring vocal biomarkers for assessing cognitive or emotional conditions rely on standardized speaking tasks, such as reading aloud, sentence repetition, tongue twisters, or speech diadochokinesis. While these methods have proven effective in extracting relevant acoustic and linguistic features, they may present challenges for individuals with cognitive impairments, who often have language disorders [45] and struggle with language production, task comprehension, or sustained attention [20]. This was notably observed in the large-scale study by Nagumo et al. [44], where participants with cognitive deficits showed reduced task performance, potentially limiting the reliability of speech-based assessments in more impaired populations.

In this context, singing emerges as a powerful and underexplored alternative. Unlike typical speech tasks, singing inherently engages motor, emotional, and cognitive systems in a highly integrated manner, offering a more natural and intuitive mode of vocal expression, even for individuals with neurocognitive disorders. Prosody, the musical part of speech which conveys mainly emotional information, can be traced back to the shared evolutionary origins of both spoken and sung communication [46].

It has recently been shown that speech and singing may have split into two systems that activate distinct neural pathways [47], as recent neuroimaging research reveals that speech and song have spatially segregated representations in the auditory cortex, suggesting that singing recruits a unique population of neurons [48]. This indicates that vocal biomarkers extracted from singing may reflect different aspects of brain function, potentially offering unprecedented sensitivity and specificity for detecting subtle changes in cognition, mood regulation, and neurological integrity. Singing requires precise coordination of the respiratory, laryngeal, and articulatory systems at a level of complexity exceeding conversational speech [49]. This increased motor control demand may reveal subtle neurological deficits that remain undetected in speech-based assessments. The sustained phonation and controlled pitch modulation inherent in singing tasks engage the vocal motor system in ways that may unmask early pathological changes [50,51].

Furthermore, singing utilizes a broader dynamic range of vocal parameters compared to speech. The greater fundamental frequency variability and formants in singing provide expanded acoustic space for detecting pathological deviations [52]. Moreover, while speech analysis must account for linguistic content, vocabulary, and grammatical complexity, singing tasks can be easily standardized and adapted across cultures and languages, and can elicit rich acoustic outputs, including variations in prosodic and spectral features [52]. Familiar songs provide standardized linguistic content while allowing assessment of vocal motor function independent of language proficiency or cognitive linguistic abilities.

The vocal features extracted from singing, combined with machine learning analysis, could provide a novel diagnostic window into brain health, complementing traditional speech-based approaches. As such, the integration of singing-based vocal biomarkers into cognitive and emotional health assessments could revolutionize the field by offering a multidimensional, engaging, and scalable tool for early detection, monitoring, and even therapeutic intervention.

Despite this growing interest and empirical support, there remains a notable lack of standardized tools specifically designed to evaluate voice features in singing. We hypothesize that singing simple melodies could provide a rich dataset of acoustic information for early cognitive and emotional assessment. Compared to standard verbal tasks, singing may enhance engagement and compliance, particularly in populations with linguistic or cognitive impairments. Singing tasks often demonstrate higher patient engagement and compliance compared to repetitive speech tasks [53], and patients with impairments in speech production sometimes preserve their ability to sing [54]. This enhanced engagement may produce more consistent and representative vocal samples, improving biomarker reliability [55]. Additionally, the emotional engagement associated with singing may activate neural networks relevant to mood and cognitive function assessment [56]. Developing standardized singing-based vocal assessments could improve the sensitivity of early screening for dementia and mental health conditions, paving the way for novel diagnostic and therapeutic applications. In this line, one promising development is the Singing Ability Assessment (SAA), an open-source test environment that captures and quantifies various aspects of human singing ability and melodic memory within an item response theory framework. The SAA assesses both melodic recall and singing accuracy, measuring the capacity to sustain long notes, a task that reflects pitch accuracy, pitch volatility, and pitch stability, as well as the ability to reproduce melodies under both rhythmic and arhythmic conditions [57]. Tools like the SAA could play a key role in advancing the field by providing standardized, scalable methods for capturing singing-based vocal biomarkers, thereby expanding the diagnostic potential of music in cognitive and mental health care.

Interestingly, the value of singing goes beyond the assessment possibilities it opens. According to the American Music Therapy Association (AMTA), music therapy is a recognized and evidence-based clinical practice that uses music to address a wide range of cognitive, emotional, psychological, and social needs [58]. As a therapeutic modality, it leverages music’s inherent capacity to modulate brain activity, enhance communication, and facilitate emotional expression. Importantly, music has a profound impact on neuroplasticity, influencing multiple levels of brain function. It stimulates neuronal connectivity, particularly between the association cortices, which are involved in higher-order cognitive processes, and supports multisensory integration and perception [58]. These effects make music therapy especially relevant for individuals experiencing cognitive decline or mental health challenges. Consequently, music-based interventions have been associated with significant improvements in quality of life and cognitive function among individuals with neurodegenerative diseases and psychiatric conditions [59,60]. In clinical contexts, music therapy is frequently used to target speech, language, memory, and executive function, particularly in populations with AD, PD, and post-stroke impairments [61]. In patients with PD and dementia, music-based interventions have shown positive effects not only on cognitive domains but also on motor function, supporting improvements in speech fluency, communication skills, and memory retrieval [62,63]. Specifically, in individuals with Alzheimer’s disease, music therapy has been found to slow the rate of cognitive decline, especially in autobiographical and episodic memory, psychomotor speed, and executive functioning, while also supporting global cognitive performance [64,65]. Compared to other treatments, singing interventions are at least as effective in delaying cognitive decline as structured, established non-pharmaceutical interventions targeting risk factors such as hypertension, obesity, smoking, depression, physical inactivity, diabetes, and social isolation [66,67]. In aphasia, a condition where it is common to have complications related to speech, music therapy has proved to improve communication, auditory comprehension, repetition, and naming [68].

Additionally, music therapy has also shown positive results in improving physiological parameters. Even a short duration of singing improved vascular function acutely in older adults, particularly in those with established or high risk of atherosclerotic cardiovascular disease. The heart rate variability, blood pressure, and oxygen saturation responses to acute singing mirror the effects of light-intensity exercise [69,70]. Similar results have been observed in swallowing disorders. Patients with presbyphagia—who often experience difficulties in respiratory control, articulation, and phonation, all closely linked to singing ability—showed improvements in both swallowing and speech functions following musical interventions [71].

Music therapy has demonstrated significant benefits for both cognitive and emotional quality of life in individuals. Adding music therapy to usual treatment or even music therapy alone was associated with clinically significant changes in well-being and health-related quality of life [67]. For instance, a recent study by Tamplin et al. [72] implemented a 20-week music therapy program for dementia patients and their family caregivers, reporting high feasibility and sustained improvements in well-being, including a notable reduction in anxiety levels. These findings underscore the therapeutic potential of music-based interventions for enhancing the quality of life in patients with neurodegenerative conditions. Music therapy has also shown efficacy in addressing mental health issues, contributing to the reduction of depressive and anxiety symptoms, and promoting emotional expression, interpersonal communication, self-esteem, and overall quality of life [73].

The mechanisms underlying these benefits are thought to involve neuroplastic changes in the brain, mostly due to continuous engagement of the brain regions related to music listening, such as the corpus callosum, prefrontal and auditory cortex, amygdala, and hippocampus [74]. These neurobiological effects position music therapy as a powerful, non-invasive intervention capable of engaging multiple cognitive and affective pathways simultaneously. Other theories propose that since music production exerts psychophysiological influence over the autonomous nervous system, singing could theoretically modulate immunological imbalances that accumulate with age [66]. In this line, Kreutz et al. [75] observed increased antibody secretion levels in participants immediately after singing and listening to choral music, supporting the idea that singing can trigger short-term immunological responses.

Taken together, these findings position music therapy (and, by extension, singing) as not only a supportive intervention but also a diagnostic and monitoring tool, capable of providing insight into cognitive functioning through observable behavioral and vocal changes.

## 6. Technical, Methodological, and Ethical Challenges

### 6.1. Data Governance and Ethical Safeguards

Vocal data are biometric identifiers, and they are thus characterized as personal data under GDPR and similar laws. The EU AI Act classifies voice-biomarker tools as high-risk AI, requiring transparency and human oversight [76]. Similarly, the Medical Device Regulation (or FDA SaMD rules in the U.S.) demands clinical evaluation, bias control, and post-market surveillance before deployment [77,78,79]. Data collection, therefore, requires explicit, revocable consent, data-minimization, transparency, and user rights to access, erase, and port their recordings [80]. Privacy-by-design measures such as on-device encryption, federated learning that leaves only aggregated model updates on the server, or splitting audio into non-identifiable feature shards are critical aspects that should be incorporated into the protocols [81,82].

In addition, it should be considered that automated outputs have an inherent error margin. For instance, Namkung et al.’s [17] stress study showed a 70% accuracy but still produced 30% false results. To avoid stigmatization, models and systems must report uncertainty, flag only “elevated risk”, and route users to human confirmation.

### 6.2. Robustness and Generalizability of Models

Current studies often rely on single-site, single-language datasets, so models overfit [83] and show reduced performance when deployed on datasets differing by language, accent, demographic factors, or recording device [84]. To avoid this, nested, site-stratified, cross-validation, and multi-center benchmarks would be required, and language-independent tasks such as sustained phonation or singing can further help [85].

Device variability is another hurdle to current approaches. Acoustic biomarkers such as shimmer, HNR, and spectral slope drift with microphone type and room acoustics, while other features such as pitch, jitter, and intensity remain more stable [86,87,88]. Researchers should incorporate calibration tones, report details about the hardware, and standardize preprocessing (resampling, denoising, normalization) to enable cross-study comparability [89,90]. On top of that, many speech-based machine learning models are adapted from general-purpose audio recognition architectures [91,92], which may lack sensitivity to subtle vocal patterns specific to clinical conditions. As an example, while several studies have found that shimmer could distinguish between healthy controls and patients with AD [42,43], when Mahon and Lachman [2] controlled for other confounding factors or conditions, such as PD (which is strongly associated with shimmer), they found that the relation between shimmer and AD markedly decreased.

### 6.3. Clinical Utility and Validation Pathway

For a vocal feature to matter in practice, it must clear three gates [93]: (1) **verification** (i.e., confirm the sensor and firmware capture audio correctly; “garbage-in, garbage-out” guardrail), (2) **analytical validation** (i.e., show that the feature reliably measures the intended construct in human subjects), and (3) **clinical validation** (i.e., demonstrate the measurement adds diagnostic or prognostic value, ideally in longitudinal cohorts that reveal predictive power years before onset).

In this line, several studies have already confirmed the clinical validity of speech analysis for differentiating individuals with AD/MCI from healthy individuals [1,2,27,42,43], or to accurately detect individuals with depression [11,12,19,20], stress [11,15,16,17,18], or Parkinson’s disease [13,21,22,28] using vocal biomarkers. Nonetheless, there is still a long way to go before the protocols can be widely validated and adopted. The translation of vocal biomarkers into clinical practice remains difficult. The main limitation lies in the limited generalizability of results, often due to factors such as sample size, language, age, or sex [12,15,42]. To address this issue, some studies have collected large and more homogeneous samples [19,27] and have cross-validated their findings [16,42] in an effort to facilitate a smoother transition into clinical application. Regarding technical limitations, the quality of voice recordings can be affected by factors such as the placement of recording devices (e.g., controlled environments vs. real-world settings) and background noise [13,21,34]. These issues are typically addressed through audio preprocessing and the use of calibration tones [16,17,18].

Future studies should be aimed at providing compelling verification and validation data to endorse the clinical value of vocal biomarkers. Developed models should publish confusion matrices and minimum viable accuracy thresholds, so clinicians understand the cost of misclassification. When these technical benchmarks align with the ethical safeguards in Section 6.1, voice-based diagnostics can progress from promising prototypes to scalable, trustworthy tools in personalized medicine.

## 7. Conclusions

Despite growing evidence supporting the utility of vocal biomarkers in detecting emotional and cognitive impairments, their integration into clinical practice remains limited. This Perspective article advocates for the systematic adoption of vocal biomarkers as a non-invasive, accessible, and cost-effective tool for early diagnosis and disease monitoring. Additionally, we propose that singing-based vocal assessments could offer a novel approach to capturing valuable acoustic markers of cognitive and emotional states. Standardizing voice-based assessments and developing AI-driven diagnostic models will be crucial in realizing the full potential of vocal biomarkers in digital health. Future research should focus on establishing reproducible methodologies, cross-cultural validation, and real-world applications to ensure scalability and clinical impact.

## Data Availability

No new data were created or analyzed in this study.

## References

[1] Fagherazzi G., Fischer A., Ismael M., Despotovic V. (2021). Voice for Health: The Use of Vocal Biomarkers from Research to Clinical Practice. Digit. Biomark..

[2] Xue C., Karjadi C., Paschalidis I.C., Au R., Kolachalama V.B. (2021). Detection of dementia on voice recordings using deep learning: A Framingham Heart Study. Alzheimer’s Res. Ther..

[3] Mahon E., Lachman M.E. (2022). Voice biomarkers as indicators of cognitive changes in middle and later adulthood. Neurobiol. Aging.

[4] Mazur A., Costantino H., Tom P., Wilson M.P., Thompson R.G. (2025). Evaluation of an AI-Based Voice Biomarker Tool to Detect Signals Consistent With Moderate to Severe Depression. Ann. Fam. Med..

[5] Hajjar I., Okafor M., Choi J.D., Moore E., Abrol A., Calhoun V.D., Goldstein F.C. (2023). Development of digital voice biomarkers and associations with cognition, cerebrospinal biomarkers, and neural representation in early Alzheimer’s disease. Alzheimer’s Dement..

[6] Pisanski K., Sorokowski P. (2021). Human Stress Detection: Cortisol Levels in Stressed Speakers Predict Voice-Based Judgments of Stress. Perception.

[7] Thomas J.A., Burkhardt H.A., Chaudhry S., Ngo A.D., Sharma S., Zhang L., Au R., Hosseini Ghomi R. (2020). Assessing the Utility of Language and Voice Biomarkers to Predict Cognitive Impairment in the Framingham Heart Study Cognitive Aging Cohort Data. J. Alzheimer’s Dis. JAD.

[8] Low D.M., Bentley K.H., Ghosh S.S. (2020). Automated assessment of psychiatric disorders using speech: A systematic review. Laryngoscope Investig. Otolaryngol..

[9] Su Z., Jiang H., Yang Y., Hou X., Su Y., Yang L. (2025). Acoustic Features for Identifying Suicide Risk in Crisis Hotline Callers: Machine Learning Approach. J. Med. Internet Res..

[10] Maffei M.F., Green J.R., Murton O., Yunusova Y., Rowe H.P., Wehbe F., Diana K., Nicholson K., Berry J.D., Connaghan K.P. (2023). Acoustic Measures of Dysphonia in Amyotrophic Lateral Sclerosis. J. Speech Lang. Hear. Res. JSLHR.

[11] Ettore E., Müller P., Hinze J., Riemenschneider M., Benoit M., Giordana B., Hurlemann R., Postin D., Lecomte A., Musiol M. (2023). Digital Phenotyping for Differential Diagnosis of Major Depressive Episode: Narrative Review. JMIR Ment. Health.

[12] Shinohara S., Toda H., Nakamura M., Omiya Y., Higuchi M., Takano T., Saito T., Tanichi M., Boku S., Mitsuyoshi S. (2020). Evaluation of the Severity of Major Depression Using a Voice Index for Emotional Arousal. Sensors.

[13] Gumus M., Koo M., Studzinski C.M., Bhan A., Robin J., Black S.E. (2024). Linguistic changes in neurodegenerative diseases relate to clinical symptoms. Front. Neurol..

[14] Robin J., Harrison J.E., Kaufman L.D., Rudzicz F., Simpson W., Yancheva M. (2020). Evaluation of Speech-Based Digital Biomarkers: Review and Recommendations. Digit. Biomark..

[15] Tang F., Chen J., Dodge H.H., Zhou J. (2022). The Joint Effects of Acoustic and Linguistic Markers for Early Identification of Mild Cognitive Impairment. Front. Digit. Health.

[16] Kappen M., van der Donckt J., Vanhollebeke G., Allaert J., Degraeve V., Madhu N., Van Hoecke S., Vanderhasselt M.A. (2022). Acoustic speech features in social comparison: How stress impacts the way you sound. Sci. Rep..

[17] Namkung J., Kim S.M., Cho W.I., Yoo S.Y., Min B., Lee S.Y., Lee J.H., Park H., Baik S., Yun J.Y. (2024). Novel Deep Learning-Based Vocal Biomarkers for Stress Detection in Koreans. Psychiatry Investig..

[18] Larsen E., Murton O., Song X., Joachim D., Watts D., Kapczinski F., Venesky L., Hurowitz G. (2024). Validating the efficacy and value proposition of mental fitness vocal biomarkers in a psychiatric population: Prospective cohort study. Front. Psychiatry.

[19] Di Y., Rahmani E., Mefford J., Wang J., Ravi V., Gorla A., Alwan A., Kendler K.S., Zhu T., Flint J. (2024). Unraveling the Associations Between Voice Pitch and Major Depressive Disorder: A Multisite Genetic Study. medRxiv Prepr. Serv. Health Sci..

[20] Ortiz K.Z., De Lira J.O., Minett T.S.C., Bertolucci P.H.F. (2024). Language impairments in Alzheimer’s disease: What changes can be found between mild and moderate stages of the disease?. Clinics.

[21] Naeem I., Ditta A., Mazhar T., Anwar M., Saeed M.M., Hamam H. (2025). Voice biomarkers as prognostic indicators for Parkinson’s disease using machine learning techniques. Sci. Rep..

[22] Wright H., Aharonson V. (2025). Vocal Feature Changes for Monitoring Parkinson’s Disease Progression—A Systematic Review. Brain Sci..

[23] Tracy J.M., Özkanca Y., Atkins D.C., Hosseini Ghomi R. (2020). Investigating voice as a biomarker: Deep phenotyping methods for early detection of Parkinson’s disease. J. Biomed. Inform..

[24] Majda-Zdancewicz E., Potulska-Chromik A., Nojszewska M., Kostera-Pruszczyk A. (2024). Speech Signal Analysis in Patients with Parkinson’s Disease, Taking into Account Phonation, Articulation, and Prosody of Speech. Appl. Sci..

[25] Menne F., Dörr F., Schräder J., Tröger J., Habel U., König A., Wagels L. (2024). The voice of depression: Speech features as biomarkers for major depressive disorder. BMC Psychiatry.

[26] Huang L., Yang H., Che Y., Yang J. (2024). Automatic speech analysis for detecting cognitive decline of older adults. Front. Public Health.

[27] Lin H., Karjadi C., Ang T.F.A., Prajakta J., McManus C., Alhanai T.W., Glass J., Au R. (2020). Identification of digital voice biomarkers for cognitive health. Explor. Med..

[28] Rohl A., Gutierrez S., Johari K., Greenlee J., Tjaden K., Roberts A. (2022). Speech dysfunction, cognition, and Parkinson’s disease. Prog. Brain Res..

[29] Kappen M., Vanderhasselt M.A., Slavich G.M. (2023). Speech as a promising biosignal in precision psychiatry. Neurosci. Biobehav. Rev..

[30] Voleti R., Liss J.M., Berisha V. (2020). A Review of Automated Speech and Language Features for Assessment of Cognitive and Thought Disorders. IEEE J. Sel. Top. Signal Process..

[31] Rickwood D.J., Coleman-Rose C.L. (2023). The effect of survey administration mode on youth mental health measures: Social desirability bias and sensitive questions. Heliyon.

[32] George S.M., Ilyas P.M. (2024). A review on speech emotion recognition: A survey, recent advances, challenges, and the influence of noise. Neurocomputing.

[33] Hashem A., Arif M., Alghamdi M. (2023). Speech emotion recognition approaches: A systematic review. Speech Commun..

[34] Zhang L., Duvvuri R., Chandra K.K.L., Nguyen T., Ghomi R.H. (2020). Automated voice biomarkers for depression symptoms using an online cross-sectional data collection initiative. Depress. Anxiety.

[35] Ding H., Lister A., Karjadi C., Au R., Lin H., Bischoff B., Hwang P.H. (2024). Detection of Mild Cognitive Impairment From Non-Semantic, Acoustic Voice Features: The Framingham Heart Study. JMIR Aging.

[36] Burke A.D., Goldfarb D. (2023). Diagnosing and Treating Alzheimer Disease During the Early Stage. J. Clin. Psychiatry.

[37] Schindler S.E. (2022). Fluid Biomarkers in Dementia Diagnosis. Continuum.

[38] García-Gutiérrez F., Alegret M., Marquié M., Muñoz N., Ortega G., Cano A., De Rojas I., García-González P., Olivé C., Puerta R. (2024). Unveiling the sound of the cognitive status: Machine Learning-based speech analysis in the Alzheimer’s disease spectrum. Alzheimer’s Res. Ther..

[39] Liu V., Smith D., Yip H. (2025). Prevalence and Treatment of Dysphonia in Parkinson’s Disease: A Cross-Sectional National Database Study. Laryngoscope Investig. Otolaryngol..

[40] Harel B., Cannizzaro M., Snyder P.J. (2004). Variability in fundamental frequency during speech in prodromal and incipient Parkinson’s disease: A longitudinal case study. Brain Cogn..

[41] Twamley E.W., Ropacki S.A., Bondi M.W. (2006). Neuropsychological and neuroimaging changes in preclinical Alzheimer’s disease. J. Int. Neuropsychol. Soc. JINS.

[42] Themistocleous C., Eckerström M., Kokkinakis D. (2020). Voice quality and speech fluency distinguish individuals with Mild Cognitive Impairment from Healthy Controls. PLoS ONE.

[43] Arslan-Sarımehmetoğlu E., Barmak E. (2025). The effect of mild-stage Alzheimer’s disease on the acoustic parameters of voice. Egypt J. Otolaryngol..

[44] Nagumo R., Zhang Y., Ogawa Y., Hosokawa M., Abe K., Ukeda T., Sumi S., Kurita S., Nakakubo S., Lee S. (2020). Automatic Detection of Cognitive Impairments through Acoustic Analysis of Speech. Curr. Alzheimer Res..

[45] Cera M.L., Ortiz K.Z., Bertolucci P.H.F., Tsujimoto T., Minett T. (2023). Speech and phonological impairment across Alzheimer’s disease severity. J. Commun. Disord..

[46] Valentova J.V., Tureček P., Varella M.A.C., Šebesta P., Mendes F.D.C., Pereira K.J., Kubicová L., Stolařová P., Havlíček J. (2019). Vocal Parameters of Speech and Singing Covary and Are Related to Vocal Attractiveness, Body Measures, and Sociosexuality: A Cross-Cultural Study. Front. Psychol..

[47] Ma W., Fiveash A., Thompson W.F. (2019). Spontaneous emergence of language-like and music-like vocalizations from an artificial protolanguage. Semiotica.

[48] Norman-Haignere S.V., Feather J., Boebinger D., Brunner P., Ritaccio A., McDermott J.H., Schalk G., Kanwisher N. (2022). A neural population selective for song in human auditory cortex. Curr. Biol. CB.

[49] Zhang Z. (2016). Respiratory Laryngeal Coordination in Airflow Conservation and Reduction of Respiratory Effort of Phonation. J. Voice Off. J. Voice Found..

[50] Behroozmand R., Khoshhal Mollasaraei Z., Nejati V., Daliri A., Fridriksson J. (2025). Vocal and articulatory speech control deficits in individuals with post-stroke aphasia. Sci. Rep..

[51] Dedry M., Maryn Y., Szmalec A., Lith-Bijl J.V., Dricot L., Desuter G. (2024). Neural Correlates of Healthy Sustained Vowel Phonation Tasks: A Systematic Review and Meta-Analysis of Neuroimaging Studies. J. Voice Off. J. Voice Found..

[52] Hansen J.H.L., Bokshi M., Khorram S. (2020). Speech variability: A cross-language study on acoustic variations of speaking versus untrained singing. J. Acoust. Soc. Am..

[53] Tamplin J., Morris M.E., Marigliani C., Baker F.A., Vogel A.P. (2019). ParkinSong: A Controlled Trial of Singing-Based Therapy for Parkinson’s Disease. Neurorehabilit. Neural Repair.

[54] Martínez-Molina N., Siponkoski S.T., Pitkäniemi A., Moisseinen N., Kuusela L., Pekkola J., Laitinen S., Särkämö E.R., Melkas S., Kleber B. (2022). Neuroanatomical correlates of speech and singing production in chronic post-stroke aphasia. Brain Commun..

[55] Alqutub A., Alqutub A., Mogharbel A.M., Awadh M.A., Sait S., Aldharrab A.S., Zagzoog F.H. (2024). Effectiveness of Singing-Based Therapy on Voice Outcomes in Parkinson’s Disease: A Systematic Review and Meta-Analysis. J. Voice Off. J. Voice Found..

[56] Hou J., Song B., Chen A.C.N., Sun C., Zhou J., Zhu H., Beauchaine T.P. (2017). Review on Neural Correlates of Emotion Regulation and Music: Implications for Emotion Dysregulation. Front. Psychol..

[57] Lichtensztejn M., Cui A.-X., Geretsegger M., Lundervold A.J., Koelsch S., Pfabigan D.M., Assmus J., Langeland E., Tabernig C., Skogseth R.E. (2024). Memory for Music (M4M) protocol for an international randomized controlled trial: Effects of individual intensive musical training based on singing in non-musicians with Alzheimer’s disease. medRxiv.

[58] Ramaswamy M., Philip J.L., Priya V., Priyadarshini S., Ramasamy M., Jeevitha G.C., Mathkor D.M., Haque S., Dabaghzadeh F., Bhattacharya P. (2024). Therapeutic use of music in neurological disorders: A concise narrative review. Heliyon.

[59] Lassner A., Siafis S., Wiese E., Leucht S., Metzner S., Wagner E., Hasan A. (2024). Evidence for music therapy and music medicine in psychiatry: Transdiagnostic meta-review of meta-analyses. BJPsych Open.

[60] Moreno-Morales C., Calero R., Moreno-Morales P., Pintado C. (2020). Music Therapy in the Treatment of Dementia: A Systematic Review and Meta-Analysis. Front. Med..

[61] Behaghel E., Zumbansen A. (2022). Singing for the Rehabilitation of Acquired Neurogenic Communication Disorders: Continuing the Evidence Dialogue with a Survey of Current Practices in Speech-Language Pathology. Healthcare.

[62] Gold C. (2016). Abstracts of the 10th European Music Therapy Conference. Nord. J. Music. Ther..

[63] Pereira A.P.S., Marinho V., Gupta D., Magalhães F., Ayres C., Teixeira S. (2019). Music Therapy and Dance as Gait Rehabilitation in Patients With Parkinson Disease: A Review of Evidence. J. Geriatr. Psychiatry Neurol..

[64] Fang R., Ye S., Huangfu J., Calimag D.P. (2017). Music therapy is a potential intervention for cognition of Alzheimer’s Disease: A mini-review. Transl. Neurodegener..

[65] Ting B., Su C.H., Chen D.T., Hsu W.T., Tsai C.L., Lin P.Y., Jingling L. (2024). The Sound of Memory: Investigating Music Therapy’s Cognitive Benefits in Patients with Dementia-A Network Meta-Analysis of Randomized Controlled Trials. J. Pers. Med..

[66] Feng L., Romero-Garcia R., Suckling J., Tan J., Larbi A., Cheah I., Wong G., Tsakok M., Lanskey B., Lim D. (2020). Effects of choral singing versus health education on cognitive decline and aging: A randomized controlled trial. Aging.

[67] McCrary J.M., Altenmüller E., Kretschmer C., Scholz D.S. (2022). Association of Music Interventions With Health-Related Quality of Life: A Systematic Review and Meta-analysis. JAMA Netw. Open.

[68] Koshimori Y., Akkunje P.S., Tjiandri E., Kowaleski J.B., Thaut M.H. (2025). Music-based interventions for nonfluent aphasia: A systematic review of randomized control trials. Ann. N. Y. Acad. Sci..

[69] Somayaji K., Frenkel M., Tabaza L., Visotcky A., Ruck T.K., Ofori E.K., Widlansky M.E., Kulinski J. (2022). Acute effects of singing on cardiovascular biomarkers. Front. Cardiovasc. Med..

[70] Somasundaram N., Mohrdieck N., Visotcky A., Kulinski J. (2025). Predictors of improvement in cardiovascular biomarkers with singing. Am. Heart J. Plus Cardiol. Res. Pract..

[71] Kim S.J., Yeo M.S., Kim S.Y., Kang S.Y. (2023). A scoping review of music-based interventions for swallowing difficulties: Implications for treating older adults with presbyphagia. Front. Med..

[72] Tamplin J., Clark I.N., Lee Y.C., Baker F.A. (2018). Remini-Sing: A Feasibility Study of Therapeutic Group Singing to Support Relationship Quality and Wellbeing for Community-Dwelling People Living With Dementia and Their Family Caregivers. Front. Med..

[73] Raglio A., Attardo L., Gontero G., Rollino S., Groppo E., Granieri E. (2015). Effects of music and music therapy on mood in neurological patients. World J. Psychiatry.

[74] Papadakakis A., Sidiropoulou K., Panagis G. (2019). Music exposure attenuates anxiety- and depression-like behaviors and increases hippocampal spine density in male rats. Behav. Brain Res..

[75] Kreutz G., Bongard S., Rohrmann S., Hodapp V., Grebe D. (2004). Effects of choir singing or listening on secretory immunoglobulin A, cortisol, and emotional state. J. Behav. Med..

[76] Busch F., Kather J.N., Johner C., Moser M., Truhn D., Adams L.C., Bressem K.K. (2024). Navigating the European Union Artificial Intelligence Act for Healthcare. npj Digit. Med..

[77] O’Brien D. (2020). Design Control, Medical Device Risk and Medical Device Regulation (MDR 2017/745): An Integrated Approach for Medical Devices.

[78] Guidance Document Medical Devices—Scope, Field of Application, Definition—Qualification and Classification of Stand Alone Software—MEDDEV 2.1/6. https://ec.europa.eu/docsroom/documents/17921/attachments/1/translations.

[79] Matts S.T., Webber C.M., Bocell F.D., Caldwell B., Chen A.L., Tarver M.E. (2022). Inclusion of patient-reported outcome instruments in US FDA medical device marketing authorizations. J. Patient-Rep. Outcomes.

[80] Regulation (EU) 2016/679 of the European Parliament and of the Council of 27 April 2016 on the Protection of Natural Persons with Regard to the Processing of Personal Data and on the Free Movement of Such Data, and Repealing Directive 95/46/EC (General Data Protection Regulation) (Text with EEA relevance). EUR-Lex. https://eur-lex.europa.eu/eli/reg/2016/679/oj.

[81] Cui Y., Li Z., Liu L., Zhang J., Liu J. Privacy-preserving Speech-based Depression Diagnosis via Federated Learning. Proceedings of the 2022 44th Annual International Conference of the IEEE Engineering in Medicine & Biology Society (EMBC).

[82] Noë A., Vaillancourt E., Zawati M.H. (2025). Verbal consent in biomedical research: Moving toward a future standard practice?. Front. Genet..

[83] Hireš M., Drotár P., Pah N.D., Ngo Q.C., Kumar D.K. (2023). On the inter-dataset generalization of machine learning approaches to Parkinson’s disease detection from voice. Int. J. Med. Inform..

[84] Wiley R.L., Schwoebel J., Shor J., Chanagala B., Caccia M., García A.M., Fisher S.D., Frasch M.G. (2025). Voice biomarkers of perinatal depression: Cross-sectional nationwide pilot study report. arXiv.

[85] Ghasemzadeh H., Hillman R.E., Mehta D.D. (2024). Toward Generalizable Machine Learning Models in Speech, Language, and Hearing Sciences: Estimating Sample Size and Reducing Overfitting. J. Speech Lang. Hear. Res. JSLHR.

[86] Fahed V.S., Doheny E.P., Busse M., Hoblyn J., Lowery M.M. (2025). Comparison of Acoustic Voice Features Derived From Mobile Devices and Studio Microphone Recordings. J. Voice Off. J. Voice Found..

[87] Bottalico P., Codino J., Cantor-Cutiva L.C., Marks K., Nudelman C.J., Skeffington J., Shrivastav R., Jackson-Menaldi M.C., Hunter E.J., Rubin A.D. (2020). Reproducibility of Voice Parameters: The Effect of Room Acoustics and Microphones. J. Voice Off. J. Voice Found..

[88] Awan S.N., Shaikh M.A., Desjardins M., Feinstein H., Abbott K.V. (2022). The effect of microphone frequency response on spectral and cepstral measures of voice: An examination of low-cost electret headset microphones. Am. J. Speech-Lang. Pathol..

[89] Brockmann-Bauser M., de Paula Soares M.F. (2023). Do We Get What We Need from Clinical Acoustic Voice Measurements?. Appl. Sci..

[90] Schuller B., Batliner A., Steidl S., Seppi D. (2011). Speech emotion recognition: Emotional models, databases, features, and algorithms. Speech Commun..

[91] Rahmatallah Y., Kemp A.S., Iyer A., Pillai L., Larson-Prior L.J., Virmani T., Prior F. (2025). Pre-trained convolutional neural networks identify Parkinson’s disease from spectrogram images of voice samples. Sci. Rep..

[92] Jeong S.M., Kim S., Lee E.C., Kim H.J. (2024). Exploring Spectrogram-Based Audio Classification for Parkinson’s Disease: A Study on Speech Classification and Qualitative Reliability Verification. Sensors.

[93] Goldsack J.C., Coravos A., Bakker J.P., Bent B., Dowling A.V., Fitzer-Attas C., Godfrey A., Godino J.G., Gujar N., Izmailova E. (2020). Verification, analytical validation, and clnical validation (V3): The foundation of determining fit-for-purpose for Biometric Monitoring Technologies (BioMeTs). npj Digit. Med..

